# Obesity and socioeconomic status in developing countries: a systematic review

**DOI:** 10.1111/j.1467-789X.2012.01017.x

**Published:** 2012-11

**Authors:** GD Dinsa, Y Goryakin, E Fumagalli, M Suhrcke

**Affiliations:** 1 Norwich Medical School, University of East AngliaNorwich, UK; 2 European Centre on Health of Societies in Transition, Department of Health Services Research and Policy, London School of Hygiene and Tropical MedicineLondon, UK; 3 UKCRC Centre for Diet and Activity Research (CEDAR), Institute of Public HealthCambridge, UK

**Keywords:** Developing countries, obesity, socioeconomic status

## Abstract

We undertook a systematic review of studies assessing the association between socioeconomic status (SES) and measured obesity in low- and middle-income countries (defined by the World Bank as countries with per capita income up to US$12,275) among children, men and women. The evidence on the subject has grown significantly since an earlier influential review was published in 2004. We find that in low-income countries or in countries with low human development index (HDI), the association between SES and obesity appears to be positive for both men and women: the more affluent and/or those with higher educational attainment tend to be more likely to be obese. However, in middle-income countries or in countries with medium HDI, the association becomes largely mixed for men and mainly negative for women. This particular shift appears to occur at an even lower level of per capita income than suggested by an influential earlier review. By contrast, obesity in children appears to be predominantly a problem of the rich in low- and middle-income countries.

## Introduction

In developed countries, obesity is widely considered a condition that affects people of lower socioeconomic status (SES) more so than those of higher SES (1). In developing countries, however, the debate continues as to whether obesity primarily affects the poor or the rich. In their comprehensive review published in 1989, Sobal and Stunkard (2) found a positive relationship between SES and obesity in developing countries: obesity appeared to be a problem predominantly of the more affluent in those countries. Subsequent reviews covering publications from 1988 through 2003 found mixed associations (3,4): McLaren (3) found that a positive association between higher SES and obesity tended to turn into an inverse association as one moved from countries with lower human development index (HDI) to countries with higher HDI (3). HDI seeks to capture the level of socioeconomic development of a country by combining three indicators – income per capita, literacy rate and life expectancy – into one composite measure.

A highly influential review of studies on the adult population in developing countries by Monteiro *et al*. (4) found mixed associations for men, but mostly inverse associations for women, concluding rather firmly that obesity was no longer solely a problem of the higher socioeconomic groups in developing countries. That review also suggested that the burden of obesity was shifting from the rich towards the poor, as one moved from countries with lower gross national income (GNI) per capita to countries with higher GNI per capita (4).

This study reviews papers published between 2004 and 2010 on the association between SES and obesity in men, women and children in developing countries. Our review adds value for several reasons. Firstly, there has been a notable growth in the number of relevant studies that merit critical synthesis since the last review had been carried out: we identified 35 studies for adults during the recent 7 years compared with 14 publications found by the last comparable review (4) over the preceding 14 years it did cover. Secondly, we use GNI per capita generated by two different methods in order to examine whether using one or the other affects the pattern of socioeconomic inequalities in obesity in relation to the level of economic development. The World Bank uses GNI per capita generated by the Atlas method in its income classification (see Discussion section for an explanation of the differences between GNI per capita generated in Atlas versus purchasing power parity [PPP] method). Thirdly, this review uses two indicators of development: GNI per capita and HDI. We do so in order to assess how far each of them acts as a factor that may account for a potentially reversing socioeconomic gradient of obesity. As an index comprising per capita income, literacy rate and life expectancy in one composite metric, it is conceivable that HDI is considered as a more appropriate indicator of ‘development’ than GNI per capita (see http://www.undp.org) and thus, possibly a more appropriate mediator of the relationship between SES and obesity. Finally, this is the first review that synthesizes the existing evidence on the association between SES and obesity among children in developing countries.

The paper is organized as follows. The next section describes the search methods and selection criteria. The third section presents the evidence on the association between SES and obesity and sheds light on how the association between SES and obesity varies by the precise SES indicator employed (i.e. education or income/wealth). We also examine in this section how the association between SES and obesity varies by either the countries’ GNI per capita or their HDI. The subsequent section provides a discussion of the results and the limitations of the study. The final section provides the general conclusions of the paper as well as recommendations for future research.

## Methods

The search strategy focused on extracting studies that empirically assessed the association between SES indicators and weight indicators in men, women and children in developing countries, using individual-level data. The sole restriction imposed on the type of study was that the underlying data had been collected on the basis of random sampling over a defined geographical unit. The main search database was MEDLINE. In addition, ECONLIT and Google scholar were searched. The search terms included *obesity*, *overweight*, *body fat*, *body weight*, *body mass index* on one hand and *socioeconomic status*, *social class*, *income*, *wealth*, *education*, *occupation*, *employment and culture* on the other. The term *‘developing countries*’ and the list of all developing countries according to the latest World Bank income classification (5) (i.e. low income <US$1,005; lower-middle income – US$1,006–3,975; and upper-middle income – US$3,976–12,275) were included to ensure the search captured all relevant countries. We refer to obesity or overweight/obesity interchangeably throughout the text because not all studies reported obesity and overweight separately. After restricting the search period to publications post-2004 (in order to avoid overlap with the previous review (4)), the final search generated 298 papers.

**Figure 1:**
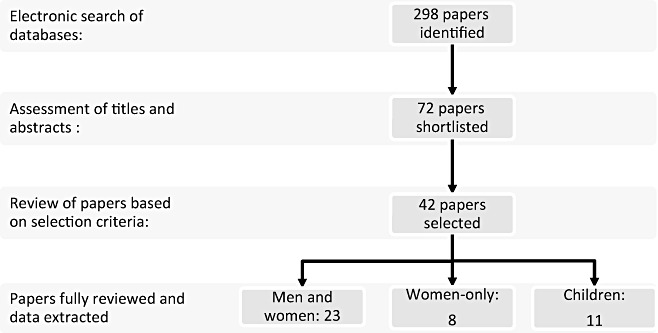
Electronic search and screening methods.

Assessing the titles and abstracts of each paper resulted in a shortlist of 72 papers. This assessment was based on whether the abstract reported on the relationship between SES and obesity and whether the country of study was a developing country, according to the definition specified earlier. We undertook further scrutiny of the full text of these 72 papers to select studies that collected data from a major city, region or nationwide (excluding small town- or community-based studies) through random sampling (to exclude convenience- or clinic-based sampling). In addition, the studies had to use measured (instead of potentially biased self-reported) weight and height data. One study on children that was undertaken in South African used dual-energy X-ray absorptiopmetry (DXA) data to measure fat mass index (FMI) and lean mass index (LMI). Finally, we generated a list of 42 papers that fulfilled the selection criteria and entered the actual review, including 23 papers on adult men and women, 8 on women-only and 11 on children (see Fig. 1 for further details of the search and screening strategy).

## Results

Four of the 42 studies we selected to review were multi-country studies, two of which – one on seven Sub-Saharan African countries (6), and another including 28 developing countries (7) – do not present data on socioeconomic inequalities by country. Hence, we could not include them in our country-specific analysis. The sample sizes for these multi-country studies were 19,992 in the Sub-Saharan Africa study and 275,704 in the study comprising 28 developing countries. Those two multi-country studies reported a positive relationship between SES and obesity on average for the sample as a whole.

The remaining two multi-country studies provide a breakdown of socioeconomic inequalities in obesity by country and are thus included in our analysis and in Table 1. These include a study undertaken in three Eastern and Central European countries (Czech Republic, Poland and Russia) (14) with data on both adult men and women, and a study covering women in three Asian countries (Bangladesh, India and Nepal) (36). Table 1 presents a summary of 33 country-specific studies on adult men and women (six country-specific reports from two multi-country studies and 27 single-country studies), while Table 2 shows a summary of 11 studies on children. In what follows, our analysis is based on studies summarized in these two tables.

**Table 1:** Association between socioeconomic status and obesity in adults in developing countries (1989–2007)

Country	Survey year	GNI per capita, PPP (USD)	GNI per capita, Atlas method (USD)	HDI	Study location	Age range	Sample size		Obesity prevalence (%)		SES indicator	Association of SES with obesity		Ref.
							Men	Women	Men	Women		Men	Women	
Seychelles	1989, 1994, 2004	11,700	6,400	0.71	National	25–64	1,525	1,818	4–15	23–34	Education, Occupation	Positive	Inverse	(8)
Vietnam	1992–2002	1,175	280	0.47	National	>18	5,512–43,500	6,470–51,065	1.2–4.4*	3.0–6.6*	Income	Positive	Positive	(9)
											Education	Inverse	Inverse	
											Occupation	Positive	Positive	
Jamaica	1993–1998	5,235	2,240	0.62	Semi-urban, around Kingston	25–74	847	1,249	8.9	33.5	Income	Positive	Positive	(10)
Brazil	1995–96	6,285	4,105	0.64	Rio de Janeiro	>20	1,413	1,866	43.9*	43.2*	Education	Inverse	Inverse	(11)
China	1998–2004	2,775	1,150	0.58	Shanghai	25–95	1,264	1,768	8.3	10	Education	None	Inverse	(12)
											Income	None	None	
Cameroon	2000	1,520	620	0.42	Yaoundé	>25	1,301	1,530	7	22	Income/wealth/asset	Positive	Positive	(13)
											Occupation	Positive	None	
Czech Republic	2002–2005	17,720	8,300	0.80	National	45–69	3,223	3,858	30	32	Education	Inverse	Inverse	(14)
Poland	2002–2005	12,200	5,800	0.78	National	45–69	4,451	4,719	27	34	Education	Inverse	Inverse	(14)
Russia	2002–2005	9,500	3,000	0.69	National	45–69	4,201	5,030	21	47	Education	None	Inverse	(14)
Mexico	2003	10,780	1,000	0.72	7 poorest states	18–65	2,576	9,071	13.4	22.5	Education, occupation, Asset	Positive	Positive	(15)
Burkina Faso	2004	960	350	0.29	Ouagadougou	>35	885	1,114	5.5	21.9	Household equipment	Positive	Positive	(16)
Iran	2004	8,590	2,210	0.65	Mazandran province	20–70	1,800	1,800	9.9	27.8	Education	Inverse	Inverse	(17)
South Africa	2004–05	8,055	4,235	0.60	Khayelitsha, Cape Town		426	549	10.1	50.3	Food availability during childhood, Adulthood income & education	None	Positive	(18)
Argentina	2004–05	9,775	4,020	0.74	Gran Chaco district	>20	204	337	13	20	Income, Education	Positive	Positive	(19)
Bulgaria	2004–06	9,260	3,600	0.72	Sofia	30–60	453	553	6.0	4.7	Income	None	Inverse	(20)
Brazil	2004–05	8,090	3,640	0.68	Pelotas, Southern Brazil	22–23	2,122	1,930	7.5	8.9	Childhood SES, Adulthood Income	Positive	Inverse	(21)
Vietnam	2005	2,100	620	0.54	Bavi district, Northern Vietnam	25–64	987	997	3.0	4.0	Income	Positive	Positive	(22)
											Education	Inverse	Positive	
											Occupation	Positive	Positive	
Iran	2005	9,140	2,570	0.66	Tabriz	≥18	132	168	18	24	Income, education	Inverse	Inverse	(23)
Philippines	2005	2,920	1,160	0.60	Metropolitan Cebu	21–22	987	819	6.1	6.5	income/wealth or asset	Positive	None	(24)
											Education	None	Inverse	
China	2005–06	4,300	1,900	0.60	Guangzhou Biobank	50–94	2,702	6,917	N/A		Childhood, early adult income, education	Positive	Inverse	(25)
Iran	2006	9,800	2,960	0.67	Razavi-Khorasan	≥30	917	1,045	7.3	15.5	Education	Positive	Positive	(26)
Benin	2005–06	1,310	560	0.43	Cotonou City	25–60	100	100	8	28	Education, occupation household amenities	Positive	Positive	(27)
Ghana	2006	1,270	590	0.45	Accra	>25	625	400	10	36	Wealth	Positive	None	(28)
Philippines	1980s–2002	1,950	805	0.56	Cebu Metropolitan	18–55		2,952		43*	Public amenities		Positive	(29)
Bolivia	1994–98	2,650	910	0.56	National	20–49		4,527		9.0–10.5	Education		Positive	(30)
India	1998–99	1,440	430	0.49	National	15–49		77,220		3	Income, education		Positive	(31)
Malaysia	1999–2000	8,075	3,400	0.69	Selangor	17–55		972		16.7	Income		Positive	(32)
											Education		Inverse	
Bangladesh	2000–04	930	380	0.49	National	15–49		242,433		4.8*	Education, Wealth		Positive	(33)
Bangladesh	2004	1,050	410	0.42	Urban	13–49		3,634		3.9	Education, Occupation		Positive	(34)
Iran	2004–06	9,230	2,580	0.70	Sistan and Baluchestan provinces	>20		888		33.5	Education		Inverse	(35)
Nepal	1996–2006	810	265	0.40	National	15–49		19,354		1.1	Income/wealth, Education		Positive	(36)
India	1998–2007	1,810	635	0.47	National	15–49		161,755		3.4	Income/wealth, Education		Positive	(36)
Bangladesh	1996–2004	855	385	0.42	National	15–49		19,211		1.4	Income/wealth, Education		Positive	(36)

* Overweight plus obese.

SES, socioeconomic status; GNI, gross national income; PPP, purchasing power parity; HDI, human development index.

**Table 2:** Association between obesity and socioeconomic status in children in developing countries (1990–2006)

Country	Survey year	GNI Per Capita, PPP (USD)	GNI per capita, Atlas method, (USD)	Study location	SES indicator	Age range	Obesity Measurement	Sample size		Obesity prevalence (%)		Association of SES with obesity	Ref.
								Boys	Girls	Boys	Girls		
Ukraine	Mid 1990s	3,000	900	Kyiv, Dneprodzerzhinsk and Mariupol cities	Social class, meat consumption and friendly neighbourhood	3-year olds	BMI ≥ 85^th^ percentile	468	415	17.7	17.7	Positive	(37)
South Africa	1990–2000	6,050	3,100	Johannesburg-Soweto	Parental education, occupation, wealth	0–10	FMI, LMI, BMI	147	134	NA	NA	Positive	(38)
Sri Lanka	2002	2,820	860	Colombo	Income, type of school	8–12	BMI	588	636	4.3	3.1	Positive	(39)
India	2002	1,710	470	Hyderabad	Composite (household possession, type of household, distance from school)	11–16	BMI	586	622	1.6	1	Positive	(40)
Vietnam	2002	1,610	430	Ho Chi Minh city	Income, household wealth, type of residence	11–16	BMI	752	752	0.9	0.3	Positive	(41)
Vietnam	2004	1,900	*540*	*Ho Chi Minh city*	Wealth, education	Adolescents	BMI	2,678		NA	NA	Positive	(42)
Guatemala	2005	4,010	*2,080*	*Quetzaltenango*	Income, type of schooling	8–10	Height for age, Weight for age, BMI		583	4.2–18.7	0.7–11.2	Positive	(43)
Vietnam	2005	2,100	620	Ho Chi Minh city	Parents’ education, wealth, occupation	4–6	Height for age, Weight for age, BMI	332	338	21.7*	11.0*	Positive	(44)
Colombia	2006	7,640	3,440	Bogota	Household assets, place of residence; Time watching TV, playing games	5–12	BMI, height-for- age	1,490	1,585	11.5*	10.7*	Positive	(45)
India	2007	2,860	1,000	South Karnataka	Time watching TV, playing games and types of diet	12–15	BMI	461	539	5.2	4.3	Positive	(46)
Iran	2006–2007	10,400	3,250	Rasht	Maternal education	12–17	BMI	N/A	2,577	N/A	5.9	Positive	(47)

* Overweight plus obese.

SES, socioeconomic status; GNI, gross national income; PPP, purchasing power parity; HDI, human development index; BMI, body mass index; FMI, fat mass index (fat mass (kg)/height (m^4^); LMI, lean mass index (lean mass (kg)/height (m^2^).

For the single-country studies, the sample size ranged from 200 in Benin to 242,433 in Bangladesh. Most of these studies employed two or more SES indicators. The two commonly employed SES indicators were education (measured by the number of years in schooling; or categorized as primary, secondary or tertiary education) and income, which is measured either by financial income or by wealth/asset indicators, generally considered as proxies for income (48). While the studies reviewed also employ occupation as an SES indicator, we focus on education and income/wealth because: (i) education and income/wealth are the two commonly used SES indicators; (ii) all of the studies that used occupation as SES indicator also used either education or income/asset or both together and (iii) the direction of the association between occupation and obesity turns out to be the same as the direction of the association between education and obesity. Hence, education may be seen as a good proxy for occupation. For children, income was defined mainly based on parental/household income, wealth or asset. A minority of child-focused studies also used the type of neighbourhood (place of residence) as a proxy for income. The sample age groups in most of the studies were 18+ for men and 15–49 (i.e. the reproductive age group) for women.

All of the studies we reviewed employed body mass index (BMI) as the indicator of ‘fatness’. Ten studies (seven for adult men and women, and three for women-only) used in addition the waist-to-hip ratio (WHR) and/or waist circumference (WC). Using WHR or WC generally resulted in a higher prevalence estimate of obesity compared with BMI (in eight out of 10 studies), but did not affect the direction and significance of the association between SES and obesity. All studies on adults but one used the common BMI cut-off points of 25–29.9 kg m^−2^ for overweight and BMI ≥ 30 kg m^−2^ for obesity. The study on China (12) used the Chinese BMI cut-off point of 28 kg m^−2^ to define obesity in addition to the standard WHO threshold.

Overall obesity prevalence in the reviewed studies ranged from 3 to 30% for men and from 1 to 50% for women (excluding the studies reporting overweight and obesity in a joint category). Low prevalence of obesity was recorded in low-income countries such as Bangladesh, India and Vietnam while high prevalence of obesity were reported in upper-middle–income countries such as Russia, Polland and Seychelles. Slightly more than half the studies (nine for adult men and women, and 15 for women) report a positive relationship between SES and obesity (excluding six studies in which the association between SES and obesity varied depending on the SES indicator employed – see Discussion later). Four studies on men and 11 studies on women reported a negative association while the findings of another four studies on men and one study on women were inconclusive.

In order to examine whether socioeconomic inequalities in obesity vary by obesity prevalence, we used the median prevalence rate (9% for men and 20% for women) as cut-off points to categorize countries into a ‘low-’ and a ‘high-’ obesity prevalence. Most of the studies that reported low-obesity prevalence (four out of six studies for men and 10 out of 14 studies for women) reported positive associations.

We also categorized studies into those based on ‘small’ and ‘large’ sample sizes, using median sample sizes (approximately 1,000 for men and 2,000 for women) as cut-off points between these two groups of studies. We found no significant difference in the association between SES and obesity among those that used a small sample and studies with a large sample.

It is important to note that all of the studies we reviewed had adjusted for age and gender (if applicable), and most of them additionally accounted for some other factors such as smoking, alcohol consumption, parity, marital status, ethnicity or place of residence. Because most studies that adjusted for more than age and gender did not provide the estimates of the correlation for just the age- and gender-adjustment, we were unable to report exclusively age- and gender-adjusted results. In Tables 1 and 2, we report the most fully adjusted results out of each study.

### Association between SES and obesity by the type of SES indicator

**Figure 2:**
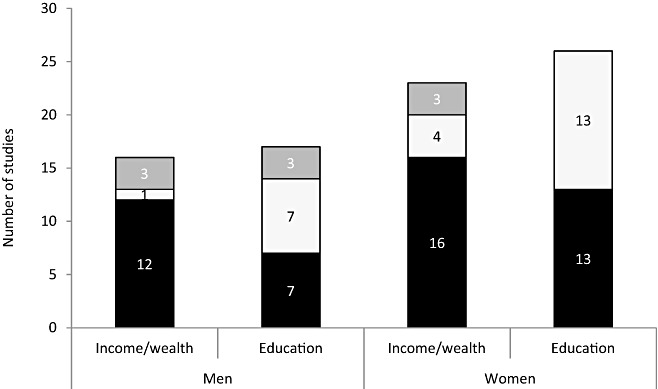
Summary of associations between socioeconomic status (SES) and obesity by main SES indicators. Black, studies with positive association; white, studies with negative association; grey, studies with no significant association.

We examined whether the type of SES indicator employed affects the pattern of socioeconomic inequalities in obesity. For men, 16 studies employed income or wealth as an SES indicator, out of which 11 reported a positive association, one reported a negative and three reported no association between income/wealth and obesity. For women, out of the 23 studies that employed income/wealth as SES indicator, 16 reported positive, four reported negative and three reported no association between income/wealth and obesity (Fig. 2). Hence, for both men and women, the majority of the studies (i.e. 69% for men and 70% for women), which used income/wealth as an SES indicator showed that the rich were more likely to be obese.

Education was used as an SES indicator by 17 studies on men, out of which seven studies reported men with more education were more likely to be obese compared with men with no (or a lower level of) education, while another seven studies reported that men with a lower level of education were more likely to be obese. The remaining three studies found no association between the level of education and obesity. Among women, out of the 26 studies that employed education as an SES indicator, 13 (13) studies found a positive (negative) association (Fig. 2).

An even more reliable judgement of whether the type of SES indicator employed affects the shape of the association between SES and obesity can be derived from studies that used both income/wealth and education as SES indicators. (The studies that did use both SES indicators, did control simultaneously for both SES indicators.) A subsample of 10 studies for men and 16 studies for women fulfilled this criterion. Among men, in seven out of these 10 studies, the direction of the association between obesity and either income/wealth or education is the same (i.e. positive in five studies, negative in one study and no association in one study). The remaining three studies find a positive association between income/wealth and obesity, but either a negative or no association between education and obesity.

Among women, in 12 out of the 16 studies that used both income/wealth and education, the choice of SES indicator does not alter the direction of the association between SES and obesity (i.e. 10 studies reported positive associations and two studies reported negative associations). For the remaining four studies, the sign of the association does depend on the SES indicator employed (positive or no relation between income/wealth and obesity, but inverse relation between education and obesity).

### Association between SES and obesity by the countries’ level of economic development

**Figure 3:**
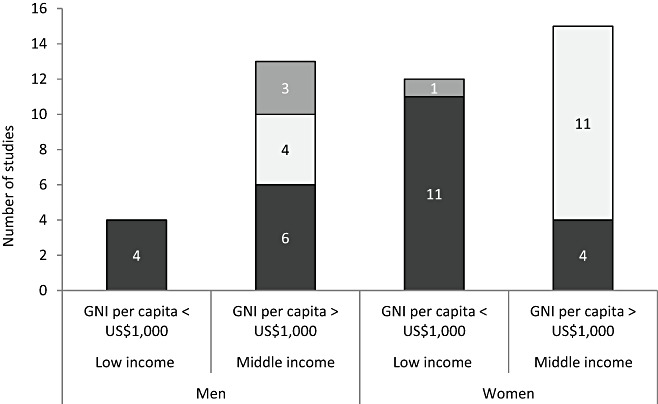
Summary of associations between socioeconomic status (SES) and obesity by gross national income. Black, studies with positive association; white, studies with negative association; grey, studies with no significant association.

Figure 3 shows that the association between SES and obesity in low-income countries is mostly positive for both men and women, excluding again the six studies in which the association between SES and obesity differs depending on the chosen SES indicator. By contrast, in the middle-income countries, the association is largely mixed for men while it is mainly negative for women. For women, out of 12 studies undertaken in low-income countries, eleven (>90%) reported that women with higher SES were more likely to be overweight/obese. On the other hand, out of 15 studies undertaken in the middle-income countries, 11(73%) reported a higher level of obesity among the lower-SES individuals. We undertook sensitivity test of these results using only studies that employed nationwide datasets and found no significant difference (see details in the Discussion section).

### Association between SES and obesity by the level of HDI – in comparison to the use of GNI per capita

**Figure 4:**
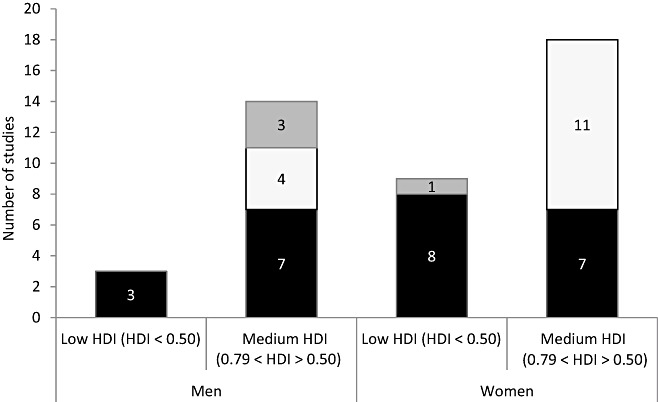
Association between socioeconomic status (SES) and obesity for men and women, in relation to human development index (HDI). Black, studies with positive association; white, studies with negative association; grey, studies with no significant association.

All but one of the 12 studies undertaken in low HDI countries – defined as countries with HDI < 0.50 – reported positive associations between SES and overweight/obesity for both men and women (Fig. 4). In countries with medium HDI (countries with HDI between 0.50 and 0.79), the association between SES and obesity is mixed for both men and women. However, a slight majority (11 out of 18) of the studies undertaken in medium-HDI countries reported a negative association between SES and obesity among women, replicating the result we found using GNI per capita as development indicator (see Figs 3 and 4 in comparison).

### Association between SES and obesity by the countries’ GNI per capita: Atlas versus PPP method

**Figure 5:**
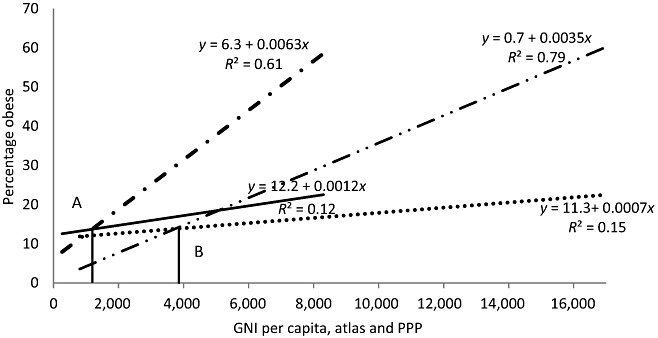
Predicted level of obesity for women by SES and GNI per capita. *Notes*: (i) With GNI per capita (Atlas method), obesity shifts from the higher-SES individuals to the lower-SES ones at point A, which corresponds to a GNI per capita of about US$ 1,000. With the PPP method, however, this shift takes place at point B, which corresponds to a GNI per capita slightly lower than US$4,000. (ii) The coefficients of GNI per capita using the Atlas method are higher than those of GNI with the PPP (0.0063 versus 0.0035 for low SES and 0.0012 versus 0.0007 for high SES), implying that the choice of GNI metric affects the strength of the relationship between obesity and income per capita. Long dash dot, low SES, Atlas; long dash dot dot, low SES, PPP; solid, high SES, Atlas; round dot, high SES, PPP; SES, socioeconomic status; GNI, gross national income; PPP, purchasing power parity.

Figure 5 plots the association between obesity (in low- and high-SES women) and GNI per capita using GNI per capita generated by both the Atlas and the PPP methods for a subsample of 14 studies that reported (i) a consistent relationship between SES and obesity irrespective of the SES indicators chosen as well as (ii) the prevalence of obesity for low- and high-SES women. GNI per capita generated by the Atlas method shows the nominal value of goods and services produced while the one calculated in PPP adjusts for local purchasing power of this income. Figure 5 shows that the choice of GNI per capita (Atlas versus PPP) can affect both the slope of the association between obesity (by SES group) and GNI per capita, and the level of per capita income at which obesity starts shifting from higher-SES women to lower-SES ones (see notes to Fig. 5). More specifically, we confirm our finding that the burden of obesity shifts from higher to lower-SES women at a GNI per capita of about US$1,000 (using the Atlas method). On the other hand, using the GNI per capita generated by the PPP method, we observe that this shift occurs at a GNI per capita of just under US$4,000 in our subsample of studies (see Fig. 5).

### Association between SES and obesity among children

The studies on children used different measures of obesity compared with those employed in the adult-related studies reported earlier. In addition to BMI, one study employed FMI, which measures fat tissue in kilograms divided by height in metres to the power of 4 [(fat mass (kg)/height(m)^4^] and LMI, which measures lean tissue divided by height in metres squared [lean tissue (kg)/height (m)^2^], while three others used height-for-age and weight-for-age. Overall, obesity prevalence varied between 1% and 18% and it was higher among boys than girls. The prevalence of obesity appears to increase with income – India and Vietnam are among countries with low prevalence while Guatemala and Ukraine are among those with relatively high obesity prevalence. In all (of the 11) studies reviewed we found a positive association between SES and obesity for both boys and girls, regardless of age, the level of GNI per capita, the level of obesity, the SES indicator chosen or the measure of fatness employed (see Table 2).

## Discussion

The purpose of this review was to take stock of the evidence on the socioeconomic inequalities in obesity in developing countries – an evidence base that has grown markedly since the last major review was published in 2004 (4). The key results of our review are as follows:

1. Within low-income countries, obesity is more prevalent among the higher-SES groups (i.e. those with higher level of income or education) than in the lower ones.
2. The pattern of socioeconomic inequalities in obesity is far more mixed in middle-income countries, particularly among men.
3. Among women, the shift in the burden of obesity from the rich to the poor occurs at a GNI per capita (calculated according to the Atlas method) of about US$1,000, and within the medium HDI range. The shift in men is considerably less visible.
4. Based on the few studies (*n* = 11) that have examined specifically the association between SES and obesity in children, the evidence unanimously depicts child obesity as being more prevalent among the affluent groups in developing countries.

The first and second results are broadly in line with Monteiro *et al*. (4), but they add value in that our conclusions are based on a considerably greater number of studies from low-income countries particularly for women. (Monteiro *et al*. included two out of 14 studies from low-income countries, while we included four out of 17 specific country-based studies for men, and 12 out of 27 for women.) The fourth result is unique to this review as no previous review had focused on inequalities in child obesity in developing countries. Reviews of high-income country studies have shown that there is generally an inverse association between SES (particularly education) and child obesity in those countries, suggesting that the shift of obesity from the rich to the poor within countries may occur at a higher level of economic development (49). Shrewsbury *et al*. reported a mixture of inverse or no association in 73% of the studies they reviewed (50). Similarly, Due *et al*. (51) found higher prevalence of overweight among adolescents from less affluent families in 21 out of 24 countries in Western Europe and North America. This demonstrates that unlike what we found in our review for developing countries, child obesity is largely a problem of poverty in developed countries. The third finding qualifies previous review evidence, in that it implies that the burden of obesity shifts at a lower level of per capita income than thought before – an issue that deserves some further elaboration:

Monteiro *et al*. had suggested that the reversal of the obesity gradient (for women) takes place at about a GNI per capita of US$2,500. Our results show that this switch-over may occur already at a considerably lower per capita income level (US$1,000). This threshold is remarkably close to the World Bank income cut-off point between low- and middle-income countries (i.e. US$1,005), using the Atlas method. A similarly clear switch-over does not appear to occur for men, or at least it occurs more slowly than in women (as was found by Monteiro *et al*.). Other recent reviews of socioeconomic inequalities in obesity have focused on high-income countries (i.e. countries with a GNI per capita >US$12,275 or an HDI > 0.80), suggesting that as countries grow into this income category, obesity even more clearly shifts to the poor within those countries, at least among women (2,3,52).

We have shown that when assessing the relationship between overall economic wealth and socioeconomic inequalities in obesity, the type of metric of the per capita GNI indicator used can greatly affect both the switch-over income threshold (unsurprisingly) as well as the slope of the association between income and obesity prevalence of both the lower- and the higher-SES group. The GNI per capita Monteiro *et al*. employed appears to be the one generated using the Atlas method (although this is not explicitly mentioned in their study), which is also the metric the World Bank has adopted for its country classification into low-, middle- and high-income categories. Using this metric, we arrive at the lower switch-over per capita income than Monteiro *et al*. If, however, we employ GNI per capita data in PPP terms, the income level at which this shift begins turns out significantly higher (about US$,4,000; see Fig. 5).

Using GNI per capita based on the Atlas method versus that based on PPP appears to particularly affect the exact relationship between national economic wealth and socioeconomic inequalities in obesity in those countries, in which the differences between incomes generated using the two methods are larger. The Atlas method reports nominal income per capita without accounting for prices of goods and services. This method does not take into account the purchasing power of the nominal income in a country. This has a significant bearing on real income particularly in poorer countries where many products (particularly food) tend to be cheaper. GNI per capita (PPP) addresses this issue by accounting for price differences among commodities (because the amount of food consumed depends not only on nominal income, but also on food prices). Under the PPP method, one US$ is considered to purchase the same quality and quantity of a commodity all over the world. Hence, using GNI per capita (PPP) for the study of obesity helps to compare differences in purchasing power or real income among countries.

### Robustness of the findings

We undertook several robustness checks to explore the robustness of our findings: (i) We examined whether results differed by sample size in the underlying study but found no significant differences. (ii) We tested whether the association between SES and obesity is affected by the type of SES indicator. We found that the choice of SES indicator (income/wealth versus education) matters in the association between SES and obesity in about 20–30% of the studies (three out 10 for men and four out of 16 for women). This is likely due to a weaker correlation between wealth and education in some developing countries, in which the underdeveloped nature of a competitive market may prevent educational investment to pay off in the labour market in the form of higher earnings and income. (iii) We have explored whether the pattern of inequalities differed by measure of fatness employed. Despite the widely recognized limitations of BMI (53,54), we do not detect differences in the patterns observed in studies that used BMI versus those using WC or WHRs. This suggests that BMI may still provide a sufficiently reliable picture of the degree of socioeconomic inequalities in overweight/obesity in developing countries, in some contrast to the finding from a US-focused study (55), which showed that the precise measure of fatness did significantly alter the association between obesity and employment. (iv) We also tested whether using national versus subnational data affects our results regarding the association between the level of GNI per capita and obesity. We found no major difference although we caution against overly generalizing this conclusion, in light of our small subsample of studies using national data (10 for women and five for men). (v) We tested whether using GNI per capita versus HDI as a development indicator matters in the association between SES and obesity – we found that there is no major difference in using either of them. (vi) We also tested whether the definition of GNI per capita matters in both the strength of the association between GNI per capita and obesity (by SES) and the level of GNI per capita where obesity starts to shift from the higher-SES to lower-SES individuals. As discussed earlier, we found that the definition of GNI per capita matters for both the level of the switch-over and the significance of the relationship between GNI per capita and obesity.

### Explaining the findings

#### Why are the poor in low-income countries ‘protected’ against obesity, and why are the rich more susceptible to it?

One obvious potential explanation for the poor in low-income countries being ‘protected’ against obesity may lie in the existence of food scarcity in those countries, which implies low/moderate food intake among the poor. In addition, the poor tend to be engaged in manual work that requires higher energy expenditure. Conversely, the observation that the rich in poorer countries are particularly susceptible to obesity could be explained by their access to surplus/excess food and a lower level of engagement in manual labour-intensive occupations (56). In addition, in some low-income countries, a larger body size might be considered as a positive status signal (57,58). Thus, in such communities, people in higher SES might prefer a larger body size (57–59). A large body size preference and its correlation with actual body size were found, for instance, by studies on Morocco (58,60) and Senegal (59).

By contrast, in many middle-income countries (or in countries with medium HDI), the issue of food shortage arguably no longer represents a common problem even for the poorest segment of the population (61). Instead, access to healthy food becomes the critical issue distinguishing the more from the less affluent. Low-calorie food (e.g. whole-grain cereals, fruits and vegetables) will likely be expensive for the poor, therefore leading to the consumption of a more energy-dense diet (62,63). For example, a recent study in rural South Africa reported that healthier diets compared with the most commonly consumed food items (e.g. whole-meal bread against white bread; brown rice against white rice; fat-free milk against full-cream milk and lean beef burger against high-fat beef burger) cost between 10 and 60% more. The authors also compared the extra cost of a recommended healthier diet to a typical South African menu and found that for an adult man, the healthier diet per day costs US$1.22 (69%) more. This study also estimated the extra cost of a healthier diet to equal US$140 per month for a household with five members, a cost that corresponds to more than 30% of the total household income for most of the population (61).

In addition to food consumption, a higher degree of urbanization and technological progress in these economies render occupations less laborious, resulting in less energy expenditure even among the poor. Obesity is by far higher among urban dwellers even in low-income countries (6), likely because of a more sedentary lifestyle. Furthermore, the poor are more susceptible to the risk of obesity, given their lower levels of education and health awareness (64). The elite in such countries, on the other hand, is more likely to be health-conscious and in a better position to invest in healthy diet and exercise in order to shield themselves from obesity (56).

Hence, the rich in poor countries would be able to afford and demand surplus food (which exposes them to obesity) while the rich in higher-income countries would likely be in a position to afford and demand a healthier diet and exercise (which prevents them from obesity). The poor in lower-income countries, on the other hand, face food shortages (which prevents them from obesity), while the poor in higher-income countries are particularly exposed to energy-dense foods (which increases their odds of becoming obese) (65). This phenomenon at the two stages of development may help explain the shift in the burden of obesity.

#### Why does the within-country shift of obesity from the rich to the poor occur faster and at earlier levels of development for women than for men?

One tentative explanation for this intriguing question may be related to the finding from research in high-income countries, suggesting there is a wage penalty associated with obesity for women (but not for men) in the labour market (66). To the extent that as countries develop, women increasingly participate in the labour force, the female wage penalty can only begin to drive the inverse SES–obesity relationship after reaching a certain level of economic development. A further potential explanation relates to the evidence that women who were nutritionally deprived as children are significantly more likely to be obese (and still socioeconomically deprived) as adults, while men who were deprived as children appear to face no greater obesity risk (18).

### Limitations

Our review synthesized the directions of the association between SES and obesity, not the strengths of these associations. A meta-analysis of the strengths of these associations using studies employing similar methodologies could, in principle, provide useful information, although it is not obvious that the underlying data and methods used across country studies could indeed be comparable enough to allow for a quantitative meta-analysis. We also caution against overly strong conclusions to be inferred from some of our findings because of the limited number of studies reviewed. These include the limited number of *nationally representative* studies (five for men and 10 for women), as opposed to the greater number of studies based on subnational samples, which render the assignment of the relevant level of per capita income somewhat arbitrary. The number of studies on children was also quite limited (*n* = 11). Moreover, it is important to bear in mind the caveat that the relationships between overweight/obesity and socioeconomic factors reported in the studies we reviewed reflect largely a simple correlation and do not allow inference about the causal nature of the (likely bi-directional) relationship.

## Conclusions

Our results shed light on the overall picture of the association between SES and obesity globally: obesity is a problem of the rich in low-income countries for both men and women, while there is a mixed picture in middle-income countries. Taken together, while on the basis of our results there is no immediate justification for a major focus on obesity prevention policies in low-income countries, obesity still does deserve considerable attention in many middle-income developing countries, both from an equity perspective – at least in women obesity is becoming disproportionately a problem of the poor already at a lower level of economic development than previously thought – and because of the sheer public health gravity of the problem across the entire population.

Future research needs to focus on some of the key questions that remain unanswered, especially the understanding of the causal structure of the interrelationship between SES and obesity in developing countries. Future research should also try to better understand why the shift in the burden of obesity from higher to lower SES occurs faster among women compared with men. More studies are also required to verify and explain the unanimously positive association between SES and child obesity in developing countries, which is very different from what is observed in developed countries. Perhaps most importantly, there is an urgent need to find out how the growing levels of obesity both among the poor and the rich in developing countries can be prevented.

## Conflict of interest statement

No conflict of interest statement.
